# Transcriptome Analysis Reveals Candidate Genes for Cold Tolerance in *Drosophila ananassae*

**DOI:** 10.3390/genes9120624

**Published:** 2018-12-12

**Authors:** Annabella Königer, Sonja Grath

**Affiliations:** Division of Evolutionary Biology, Faculty of Biology, LMU Munich, Grosshaderner Str. 2, 82152 Planegg-Martinsried, Germany; koeniger@bio.lmu.de

**Keywords:** chill coma recovery time, cold tolerance, adaptation, *Drosophila ananassae*, RNA sequencing, heat shock proteins, actin polymerization, apoptosis

## Abstract

Coping with daily and seasonal temperature fluctuations is a key adaptive process for species to colonize temperate regions all over the globe. Over the past 18,000 years, the tropical species *Drosophila ananassae* expanded its home range from tropical regions in Southeast Asia to more temperate regions. Phenotypic assays of chill coma recovery time (CCRT) together with previously published population genetic data suggest that only a small number of genes underlie improved cold hardiness in the cold-adapted populations. We used high-throughput RNA sequencing to analyze differential gene expression before and after exposure to a cold shock in cold-tolerant lines (those with fast chill coma recovery, CCR) and cold-sensitive lines (slow CCR) from a population originating from Bangkok, Thailand (the ancestral species range). We identified two candidate genes with a significant interaction between cold tolerance and cold shock treatment: *GF14647* and *GF15058*. Further, our data suggest that selection for increased cold tolerance did not operate through the increased activity of heat shock proteins, but more likely through the stabilization of the actin cytoskeleton and a delayed onset of apoptosis.

## 1. Introduction

When organisms are faced with new environmental conditions, such as those caused by climate change or range expansion, they must adapt in order to survive. From an evolutionary perspective, studying how species are formed and how they adapt to their environment are central questions. Local adaptation is an evolutionary process by which individuals have their highest fitness in their local environment by means of natural selection. This means that a specific trait will be selected in a local population as a consequence of local habitat characteristics. Species having a worldwide distribution are excellent models for studying local adaptation. One example of local adaptation that has been studied in detail is adaptation to cold. In ectothermic organisms, body temperature mainly follows the external environment, and thermal regulation mechanisms are limited. Therefore, the geographical distribution and abundance of these species strongly depends on their ability to cope with local temperatures. Insects, with their high diversity and ability to colonize almost all of the ecosystems on Earth, are an excellent group of ectotherms to study cold adaptation (as reviewed by Overgaard and MacMillan in [1]).

Species that are able to remain active at sub-zero temperatures rely on one of two strategies to survive the cold: they either tolerate the formation of ice crystals in their body fluids (freeze-tolerant insects, [2]) or they avoid freezing by supercooling (freeze-avoiding insects, [3]). However, many insect species evolved and diversified at much higher temperatures [4,5], and are susceptible to chilling injuries when they are exposed to a cold environment (chill-susceptible insects [6]). Being exposed to their critical thermal minimum, chill-susceptible species enter a stage of chill coma. At this stage, they are not able to move, feed, mate, or escape predators, but are able to recover if the time of exposure and the temperature of the cold shock do not exceed a certain threshold [7]. Chill coma is accompanied by a disruption in water balance and ion homeostasis, the loss of neuromuscular coordination, and disruption of membrane fluidity ([1,8,9]). 

A prominent example for chill-susceptible insects is species from the genus *Drosophila*. Drosophilids are vinegar flies that have been an important model system for biological studies since the early 1900s. There is a growing body of literature that recognizes the complexity of thermotolerance in *Drosophila* spp. e.g., Telonis-Scott et al. [10], von Heckel et al. [11], Gerken et al. [12], Poupardin et al. [13] and other insect species e.g., Des Marteaux et al. [14,15], Torson et al. [16], Ragland et al. [17], Kvist et al. [18], see Overgaard and MacMillan in [1] for a general review on insect chill tolerance]. In particular, *Drosophila* spp. having a worldwide distribution are excellent models for studying temperature adaptation. Using a model system such as *Drosophila* for studying adaptation to cold has arguably many advantages over other ectotherms; for example, it has a small genome that is sequenced and well annotated. While *Drosophila melanogaster* is by far the best-studied species of the genus, other species have recently gained relevance in evolutionary studies. One example is *Drosophila ananassae*, which is a species that is mainly found in human-inhabited areas in tropical and subtropical regions, and has a quasi-cosmopolitan distribution, meaning it is only absent in some cold areas [19]. The species is most abundant in Southeast Asia, where it most likely originated [20]. 

One way of assessing cold tolerance plasticity for drosophilids in the laboratory is by measuring chill coma recovery time (CCRT, but see Andersen and colleagues [9] for a discussion of various other measures) which is defined as the time it takes for a fly to recover (i.e., to have the ability to stand) when brought back to room temperature following a cold-induced coma [7]. Using this method, it has been shown that *Drosophila* spp. with a tropical and subtropical distribution take longer to recover from a cold shock than species from temperate regions [9,21,22]. Furthermore, CCRT follows latitudinal clines within species as shown for *Drosophila subobscura* [23] and *D. ananassae* [24], which is consistent with local adaptation. 

Previously, we conducted a preliminary analysis of CCRT in two *D. ananassae* populations as a proxy to investigate adaptation to their local environment [25]. We assayed flies from a tropical population of the ancestral species range in Bangkok (Thailand) and flies from a derived, temperate population in Kathmandu (Nepal). The flies from Kathmandu recovered faster than the flies from Bangkok, which is consistent with adaptation to the colder environment. Interestingly, within the Bangkok population that shows no within-population structure and likely corresponds to the ancestral species range [20,26], there were two phenotypic groups: cold-tolerant strains (fast CCR) and cold-sensitive strains (slow CCR). The bimodal distribution of CCRT among Bangkok strains could suggest that variation at only a few genes underlie the observed difference in cold tolerance within this population. This observation is of great importance, as we know from previous studies in *D. melanogaster* that CCRT is a highly polygenic trait that involves many genes of small effect and many epistatic interactions among genes [9,11,27]. The cold-tolerant *D. ananassae* strains from Bangkok had a CCRT that was very similar to the CCRT for strains from Kathmandu, suggesting that the variation is ecologically relevant. Further, selection in the derived population may have used standing variation present in the ancestral population, which was selected for when colonizing more temperate habitats. Therefore, the identification of these genetic variants may uncover candidate genes for cold tolerance without possibly the confounding effects of population divergence.

In the present study, we first performed phenotypic assays to get a clear picture of CCRT in the two *D. ananassae* populations from Bangkok and Thailand. Second, we conducted a transcriptome analysis on differential gene expression among cold-tolerant and cold-sensitive fly strains for the Bangkok population in response to a cold shock. The transcriptome can be seen as link between genotype and phenotype, and may serve as a proxy to gain insight into cold adaptation and uncover genes that have undergone regulatory evolution. Such genes might help in achieving greater cold tolerance in different species or strains. We expected to see effects at the regulatory level in two possible situations: (i) before the cold shock, where there might be an advantageous transcriptional basis before the flies are subjected to cold, and (ii) during the recovery phase, where there might be an improvement in the fly’s response to cold stress, leading to a faster recovery. Therefore, we extracted RNA from male flies at three different time points: (1) before the cold shock at room temperature (control), (2) at 15 min after the cold shock, and (3) at 90 min after the cold shock. The two time points in the recovery phase were chosen to make our study comparable to a recent study performed to uncover the regulatory differences between a temperate and tropical population of *D. melanogaster* [11]. *D. melanogaster* and *D. ananassae* both belong to the Melanogaster group, and shared a common ancestor about 15–20 million years ago [28,29]. Having comparable data from both species and different populations allowed us to study the genetic basis of CCRT in two independent phylogenetic lineages (Melanogaster subgroup and Ananassae subgroup), and to uncover common evolutionary patterns among species that have expanded their ranges. Third, the preliminary characterization of differentially expressed genes in *D. ananassae* allowed us to identify candidate genes with an adaptive potential for cold tolerance.

## 2. Materials and Methods 

### 2.1. Fly Strains

We used eight fly strains that were collected in 2002 in Bangkok (Thailand), and three fly strains that were collected in 2000 in Kathmandu (Nepal). The strains were established as isofemale strains (see Das and colleagues in [20] for further details on the populations). All of the flies were kept at low density at a constant temperature (22 ± 1 °C) and at a 14:10 h light:dark cycle. The flies were raised in 50-mL vials containing custom-made food medium. In brief, the medium was based on cornmeal, sugar beet molasses, and dry yeast, containing agar-agar for medium consistency and propionic acid and nipagin as preservatives (see von Heckel and colleagues in [11]).

### 2.2. Chill Coma Recovery Assays

Chill coma recovery time was measured for all 11 fly strains for male and female flies separately. At the age of zero to two days, the flies were sex-separated under light CO_2_ anesthesia, and 10 individuals from the same strain were collected into a 50-mL vial containing 10 mL of food medium. At the age of four to six days, the flies were transferred without anesthesia into new vials without food. For the cold shock, the vials were placed in an ice water bath (0 ± 0.5 °C) for exactly three hours. Subsequently, the vials were brought back to room temperature (22 ± 1 °C), and CCRT was monitored in two-minute intervals for the duration of 90 min. Flies that were not standing after 90 min, but moved after tabbing the vial, were assigned a recovery time of 92 min. Flies that died during the experiment accounted for less then 1% of the tested flies, and were excluded from the analysis. 

### 2.3. RNA Extraction and RNA Sequencing

RNA extractions were performed for the eight fly strains from Bangkok on two biological replicates per strain at three different time points: before the cold shock at room temperature, 15 min after the cold shock, and 90 min after the cold shock (Figure S1). For each sample, eight whole male flies at four to six days of age were pooled. RNA was extracted using the MasterPure RNA Purification Kit (Epicentre, Madison, WI, USA) following the manufacturer’s protocol without DNAseI digestion. The cold shock was performed as described in the Section 2.2. RNA quality was confirmed with a NanoDrop© (ND 1000, VWR International, Radnor, PA, USA) and a bioanalyzer (Bioanalyzer 2001, Agilent Technologies, Santa Clara, CA, USA, provided by the LMU genomics service unit), and then sent to an external sequencing facility (GATC, Konstanz, Germany) which carried out poly(A) enrichment, fragmentation by sonication, 3′-cDNA synthesis, and single-end sequencing of 50 bp reads on seven lanes of a HiSeq 2500 Illumina (San Diego, CA, USA) sequencer.

### 2.4. Read Mapping and Differential Gene Expression Analysis

The raw reads were mapped to the *D. ananassae* transcriptome (dana_r1.05_FB2016_01, including non-coding RNAs) using the annotation of FlyBase release 1.05 [30]. Mapping of the raw reads was done with NextGenMap (version 0.4.12) [31], which has been shown to produce reliable alignments in *Drosophila* [11,32]. Differentially expressed genes were called with DESeq2 (version 1.16.1), [33] as implemented in R (version 3.3.0) [34]. A Wald test was used to test for significance in log2 fold changes. *p*-values were subsequently corrected for multiple testing according to Benjamini–Hochberg [35], and the false discovery rate (FDR) was set to 5%, i.e., all of the genes with a corrected *p*-value ≤ 0.05 were reported as differentially expressed. DESeq2 corrects for library size and library composition using size factors that are calculated based on the given expression data. The geometric mean is calculated for each gene across all of the samples. The read counts for a gene in each sample are then divided by this mean. The median of these ratios in a given sample is the size factor for that sample. Further, DESeq2 uses a generalized linear model and shrinkage estimators for dispersion and fold change, and thereby accounts for genes with low read counts and high dispersion. We used a two-factor design plus an interaction term (~phenotype + time point + phenotype:time point) to analyze the effects of phenotype (with two levels: slow CCR and fast CCR) and time point (with three levels: control at room temperature, 15 min, and 90 min after the cold shock) on gene-expression levels.

### 2.5. Gene Ontology Term Enrichment Analysis

We used the Database for Annotation, Visualization and Integrated Discovery (DAVID) (version 6.8) [36] to get an overview of Gene Ontology (GO) terms that are associated with lists of differentially expressed genes. Enrichment analysis was performed against the background of all of the annotated Flybase gene IDs [30] for three categories: biological process (BP), molecular function (MF), and cellular component (CC), with a minimum count of two genes per category to be reported. Gene Ontology terms were counted as significant with an Expression Analysis Systematic Explorer (EASE) score of 0.05 after multiple testing correction, according to Benjamini–Hochberg [35]. Lists with significant GO terms were then submitted to a web server that reduces and visualizes Gene Ontology terms REVIGO [37] to remove redundant terms.

### 2.6. Comparison with Drosophila melanogaster

Previously, von Heckel et al. [11] used RNA sequencing to analyze differential gene expression before and at 15 min and 90 min after a cold shock in cold-sensitive *D. melanogaster* populations with slow CCR (Africa) and cold-tolerant populations with fast CCR (Europe). It needs to be noted that in their study, they applied a cold shock of seven hours, whereas we applied a cold shock with a duration of three hours only. There are two reasons for this deviation in the experimental protocol: first, using a preliminary test, we found that the tropical species *D. ananassae* is much more cold sensitive, and the flies do not survive seven hours at 0 °C. Second, a three-hour exposure to 0 °C for *D. ananassae* leads to similar CCRT as a seven-hour exposure to 0 °C for *D. melanogaster*. Thus, synchronizing CCRT allowed us to create a dataset that can be used to compare gene expression in response to a cold shock across both species. To do so, we used mapped *D. melanogaster* reads that were kindly provided by von Heckel et al., and analyzed them with the same DESeq2 model as the *D. ananassae* reads. 

## 3. Results

### 3.1. Phenotypic Differences in Cold Tolerance as Measured by Chill Coma Recovery Time

**Figure 1:**
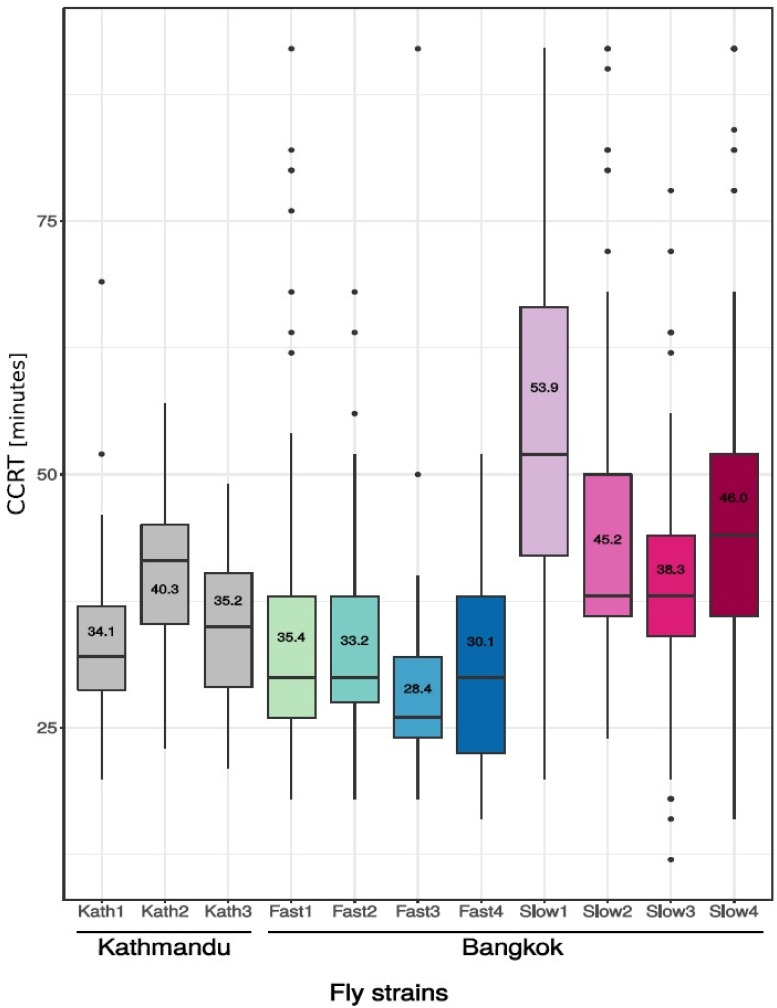
Chill coma recovery time (CCRT) after a cold shock of three hours at 0 °C in four to six-day-old male flies of two populations of *Drosophila ananassae*: Kathmandu, Nepal (grey) and Bangkok, Thailand (cold-tolerant strains are shown in shades of blue; cold-sensitive strains are shown in shades of red). Data for the individual flies is given in Additional File 3. *Y*-axis: CCRT in minutes. *X*-axis: fly strain.

Chill coma recovery time was measured after a cold shock of three hours in male flies of eight isofemale fly strains from a population that originated from Bangkok, and three fly strains from Kathmandu (Figure 1, Additional File 3). Across all of the tested strains, CCRT ranged from a minimum of 12 min to a maximum of >92 min. The effect of the fly strain on CCRT was highly significant (Kruskal–Wallis test, *p* < 2.2 × 10^−16^). Within the Bangkok population, there existed strains with fast CCR (cold-tolerant) and slow CCR (cold-sensitive). The mean CCRT of all of the cold-tolerant strains combined was 31.70 min, and of all cold-sensitive strains combined was 45.86 min. The difference between the means was highly significant (Mann–Whitney U-test, *p* < 2.2 × 10^−16^).

### 3.2. Transcriptome Overview

**Figure 2:**
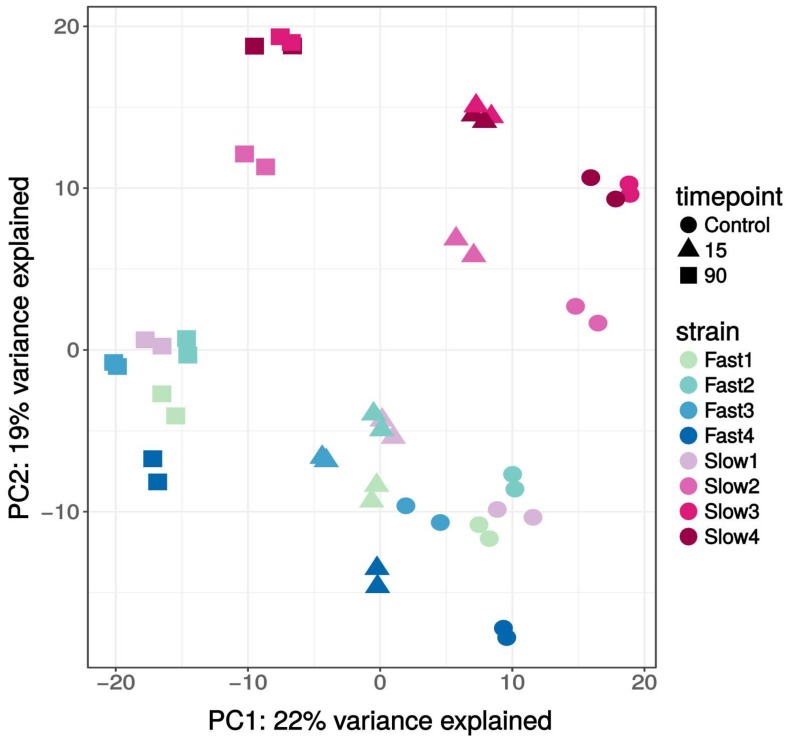
Principal component analysis (PCA) based on the 500 most variable genes. See Additional File 6 (Figure S3) for PCA plots with 250 genes and all of the genes in the dataset. Cold-tolerant strains (fast CCR) are shown in shades of blue; cold-sensitive strains (slow CCR) are shown in shades of red.

We obtained transcriptome data from 48 complementary DNA (cDNA) libraries with an average of 49 Mio. reads and Phred quality scores above 30. The sequence quality was confirmed with FastQC (version 0.11.4) [38]. The proportion of reads that could be mapped to the reference was >94% for each sample. A principal component analysis (PCA) based on the 500 most variable genes revealed tight clustering of the biological replicates (Figure 2). The first principal component accounts for 22% of the variance, and clearly separated the three time points. The second principal component accounted for 19% of the variance, and separated the fly strains from each other. Note that the data grouped according to strain and time point irrespective of subdividing the data (see also Additional File 6 for PCA plots based on the most variable genes and based on all of the genes).

### 3.3. Analysis of Differential Gene Expression

DESeq2 [33] was used to determine the differential gene expression at room temperature and 15 min and 90 min after a three-hour cold shock among cold-tolerant and cold-sensitive fly strains. Of the 14,365 protein-coding genes annotated in FlyBase release 1.05, 14,250 (99.2%) had at least one read in at least one of the libraries. For 13,562 genes, at least one read could be mapped in all of the libraries. The numbers of differentially expressed genes in each category are shown in Table 1 for a FDR of 5%. 

### 3.4. Expression Differences before the Cold Shock

As a reference for analysis of differential gene expression in response to a cold shock, we extracted RNA at room temperature from fly strains of both phenotypes. When raised under common garden conditions without being subjected to cold stress, 3.87% of all of the genes had higher expression in the cold-tolerant strains than in the cold-sensitive strains, and about the same proportion (3.91%) had higher expression in the cold-sensitive strains than in the cold-tolerant strains (Table 1). After multiple testing correction, there was no significant GO enrichment in either of the two categories (Additional File 4: Tables S1 and S2). 

### 3.5. Expression Differences in the Recovery Phase

To uncover the underlying genetic regulatory basis of the phenotypic difference between the cold-tolerant and cold-sensitive strains within a single population, we chose two distinct time points in the recovery phase: 15 min (i.e., in the early recovery phase before the first fly stands up) and 90 min (i.e., in the late recovery phase when almost all flies are recovered). 

First, we present the results for the comparison *control* versus *15 min after the cold shock*. There were more genes differentially expressed in the cold-sensitive phenotype than in the cold-tolerant phenotype (178 genes versus 57 genes, Table 1, Additional File 4). About twice as many genes are upregulated, and roughly 10 times more genes are downregulated in the cold-sensitive phenotype compared to the cold-tolerant phenotype (Table 1). No genes were significantly upregulated in one phenotype and downregulated in the other one. After multiple testing correction, there was no significant GO enrichment in either of the two phenotypes among upregulated or downregulated genes (Additional File 4: Tables S3, S4, S6, and S7). To identify the general characteristics of the response to a cold shock at this time point, we investigated genes that were significantly differentially expressed in the same direction in both phenotypes. In the set of upregulated genes, 33 genes overlapped between the cold-sensitive phenotype and the cold-tolerant phenotype. They were enriched in 10 molecular function categories related to nucleotide binding (Additional File 4, Table S5). The gene with the highest fold change was *Hsp70* in both phenotypes. This is in line with previous studies on *D. melanogaster* [11]. In the set of downregulated genes, six genes overlapped between the cold-sensitive phenotype and the cold-tolerant phenotype (Additional File 4, Tables S5 and S6). Among them are the orthologs of *odd skipped* (a transcription factor [39]), *granny smith,* which has metallopeptidase activity and is involved in proteolysis [40], and *mulet,* which is involved in spermatoid development [41]. Among the five genes that are downregulated exclusively in the cold-tolerant fly strains is the ortholog of *Senescence marker protein-30* (*smp-30*). In *D. melanogaster*, *smp-30* transcription levels have been reported to increase following cold acclimation, and the protein is thought to play a role in the cytosolic maintenance of Ca^2+^ levels [42]. Overall, we see a downregulation of *smp-30* at both time points after the cold shock in *D. ananassae*, as well as in our reanalysis of the *D. melanogaster* data.

Second, we present the results for the comparison *control* versus *90 min after the cold shock*. There were 2072 differentially expressed genes in the cold-sensitive phenotype, and 10,749 genes differentially expressed in the cold-tolerant phenotype (Table 1, Additional File 4). No gene was significantly upregulated in one phenotype and downregulated in the other one. In contrast to the 15 min time point, the same proportion of genes (about 8%) was upregulated in both phenotypes (Table 1). Among all of the upregulated genes, 888 were shared between the phenotypes; 198 genes were upregulated only in the cold-sensitive strains, and 208 genes were upregulated only in the cold-tolerant strains. Again, the gene with the highest fold change in the expression in both phenotypes was *Hsp70*.

**Figure 3:**
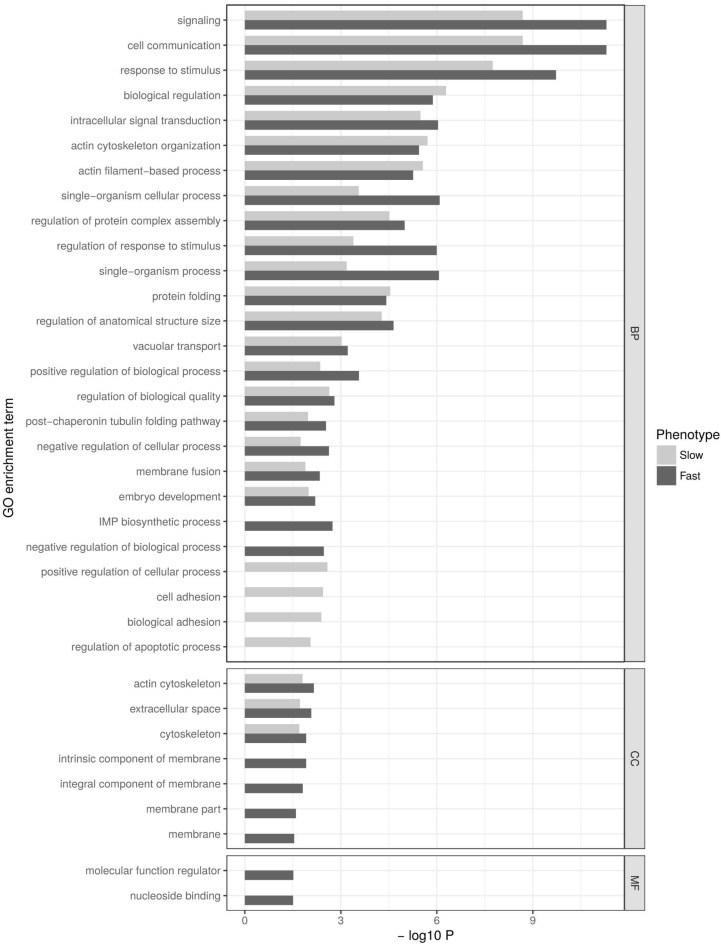
Gene Ontology (GO) enrichment analysis for genes that were significantly upregulated (*p* < 0.05) at 90 min after the cold shock in cold-sensitive (slow CCR, light bars) and cold-tolerant fly strains (fast CCR, dark bars). Gene Ontology terms are plotted according to the significance of their enrichment (−log10 *p*-value after Benjamini–Hochberg correction). Terms in three different categories are shown: biological process (BP), cellular component (CC), and molecular function (MF).

After removing redundant GO terms, the set of genes that were upregulated in both phenotypes was significantly enriched for several categories (Figure 3). There are 23 terms that are common between the phenotypes, among which the strongest enrichment was seen in BP terms related to signal reception and response, such as “signaling”, “cell communication”, and “response to stimulus” (Additional File 4: Tables S9 and S10). Terms that were exclusively enriched in the cold-tolerant phenotype included four CC terms related to “membrane” (Additional File 4: Table S9), whereas terms that were exclusively enriched in the cold-sensitive phenotype included “cell adhesion”, but also “regulation of apoptotic process”. Enrichment in the term “regulation of apoptotic process” was about fivefold (Additional File 4: Table S10) and driven by two genes (*CIAPIN1* and *BI-1*) that are apoptosis inhibitors [43,44], and by two genes (*GF24126* and *Dronc)* that positively regulate apoptosis [43,45].

**Figure 4:**
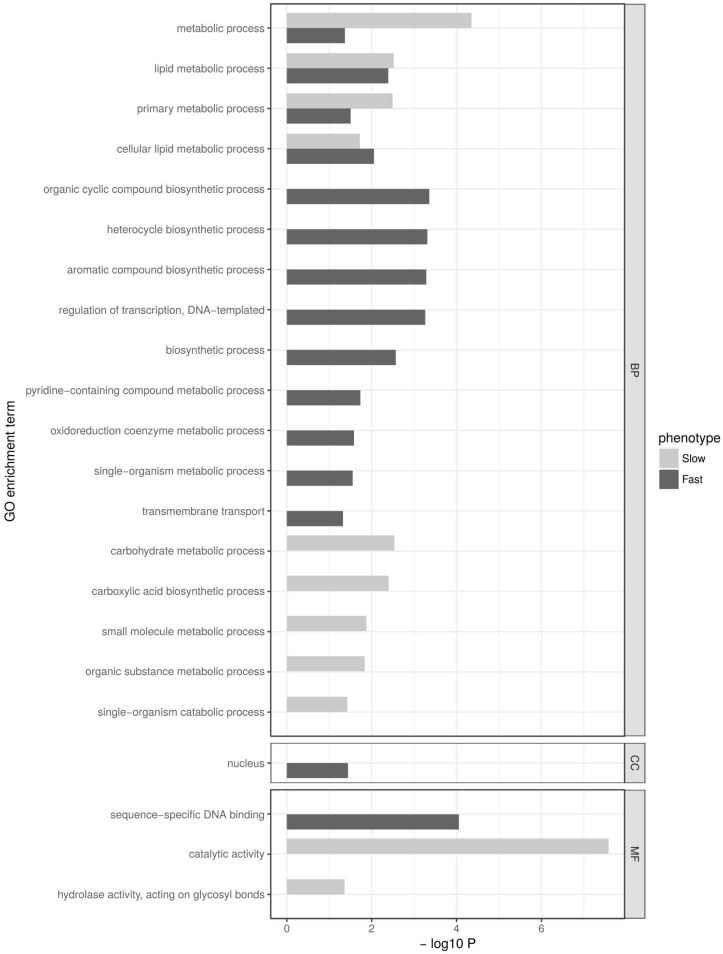
Gene Ontology enrichment analysis for genes that were significantly downregulated (*p* < 0.05) at 90 min after the cold shock in cold-sensitive fly strains (slow CCR, light bars) and cold-tolerant fly strains (fast CCR, dark bars). Gene Ontology terms are plotted according to the significance of their enrichment (−log10 *p*-value after Benjamini–Hochberg correction). Terms in three different categories are shown: biological process (BP), cellular component (CC) and molecular function (MF).

Similar to the 15 min time point, more genes were downregulated in the cold-sensitive strains (7.1%) than in the cold-tolerant strains (4.8%) at 90 min after the cold shock (Table 1). Among all of the downregulated genes, 489 were shared between the phenotypes, 163 were only downregulated in the fast strains, and 498 genes were only downregulated in the cold-sensitive strains (Additional File 4: Tables S9 and S10). Again, after removing redundant GO terms, the downregulated genes of both phenotypes were significantly enriched for several categories, most of which included terms related to metabolism (Figure 4). Four of them were commonly enriched in both phenotypes (Additional File 4: Tables S12 and S13). Terms that were exclusively enriched in the cold-tolerant phenotype include further “metabolic process” terms, but also terms related to “biosynthetic process” (Additional File 4: Table S12), whereas terms that were exclusively enriched in the cold-sensitive phenotype include terms related to “catabolic process” (Additional File 4: Table S13).

### 3.6. Comparison with Drosophila melanogaster

For comparison with *D. melanogaster*, we re-analyzed the mapped read counts obtained from von Heckel et al. [11] with the same DESeq2 model as the *D. ananassae* data (Table 2). Compared to *D. ananassae*, we saw a higher proportion of genes with a significant interaction between phenotype and time point, a higher proportion of genes that responded to the cold shock, and about four times more genes than those were differentially expressed at room temperature between the two phenotypes (Table 1 and Table 2). This finding is in line with our expectations, since the two phenotypes in *D. melanogaster* are derived from two distinct populations (Africa and Europe) that are known to show genetic differentiation [46], and the two phenotypes in *D. ananassae* are from a single population.

### 3.7. Expression of Heat-Shock Proteins

**Figure 5:**
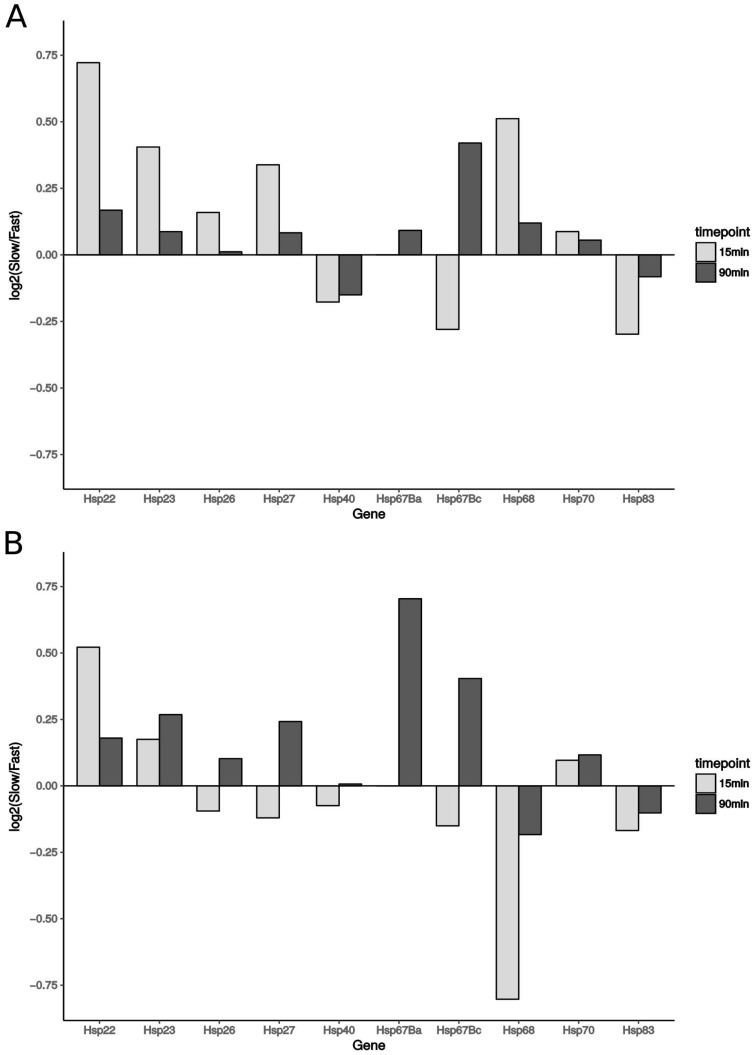
Comparison of transcriptional log2 fold changes of heat-shock proteins (Hsps) in cold-sensitive (slow CCR) and cold-tolerant (fast CCR) fly strains at 15 min (light bars) and 90 min (dark bars) in *Drosophila ananassae* (**A**) and *Drosophila melanogaster* (**B**). Bars represent the slow: fast log2 ratios of the log2 fold changes for each gene. A stronger transcriptional response in the cold-sensitive strains compared to the cold-tolerant strains can be seen as a positive bar, a stronger transcriptional response in the cold-tolerant strains compared to the cold-sensitive strains can be seen as a negative bar. Log2 fold changes for each gene and sample can be found in Additional File 4.

Heat-shock proteins (Hsps) were the genes with the strongest transcriptional response in both phenotypic groups and at both time points after the cold shock. Overall, we identified *D. ananassae* orthologs of the following 10 Hsps as cold-inducible: *Hsp22*, *Hsp23*, *Hsp26*, *Hsp27*, *Hsp40*, *Hsp67Ba*, *Hsp67Bc*, *Hsp68*, *Hsp83,* and *Hsp70*. For most of the Hsps, the transcriptional response after the cold shock was stronger in the cold-sensitive phenotype then in the cold-tolerant phenotype (Figure 5A). The only exceptions were *Hsp40*, *Hsp67Bc,* and *Hsp83*, which showed a stronger effect in the cold-tolerant phenotype. We then looked at the expression of Hsps in *D. melanogaster*, and found that the expression patterns are very similar among the species, except for *Hsp68*, which in *D. ananassae* showed stronger upregulation in the cold-sensitive phenotype than in the cold-tolerant phenotype. However, in *D. melanogaster, Hsp68* showed stronger upregulation in the cold-tolerant phenotype (from Europe) than in the cold-sensitive phenotype (from Africa) (Figure 5B).

### 3.8. Identification of Candidate Genes Involved in Cold Tolerance in Drosophila ananassae

To identify the candidate genes that might underlie the phenotypic difference in CCRT for populations of *D. ananassae*, we applied two different approaches. (1) First, we used an interaction term within our DESeq2 formula to identify genes that respond to the cold shock in a phenotype-specific way. (2) Second, we aimed to identify those genes that could contribute to increased cold tolerance, because they are already expressed at higher levels at room temperature.

With the first approach, we identified three genes with a significant interaction between phenotype and the time point 90 min after the cold shock (Table 1). As it is possible that the significance is driven by only a few strains or replicates within each of the phenotypes, we visualized the counts in each sample to verify the candidates. One of the three genes (*GF25091)* turned out to be such a false-positive case. The significance seemed to be driven by only four samples with a high number of read counts (Figure S2E). Therefore, we excluded that gene from our list of candidates. Thus, two candidate genes with significant interaction remained: *GF14647* and *GF15058* (Table 3). 

The first candidate gene (*GF14647*) was higher expressed at room temperature in the cold-sensitive strains compared to the cold-tolerant strains. In the cold-tolerant strains, expression increased at both time points after the cold shock. In the cold-sensitive strains, expression only increased at the 15 min time point, but decreased at 90 min to control level (Figure S2A). The *D. melanogaster* ortholog of *GF14647* is *CG10621*. In our analysis of the *D. melanogaster* data, we see a different pattern: in both *D. melanogaster* phenotypes (i.e., populations), *CG10621* was downregulated after the cold shock. The downregulation was stronger in the cold-tolerant European (fast CCR) strains, in which expression levels at room temperature were higher (Figure S2B). The function of the *CG10621* protein has not been characterized yet, but it contains conserved domains that belong to the “selenocysteine methyltransferase activity” family [47], suggesting that it catalyzes the methylation of selenocysteine into Se-methylselenocysteine. The second candidate gene (*GF15058*) was not differentially expressed at room temperature between the phenotypes. In the cold-tolerant strains, expression decreased at the 15 min time point, and increased at the 90 min time point. In the cold-sensitive strains, downregulation at 15 min after cold shock was stronger, and the expression levels did not increase at the 90 min time point (Figure S2C). The *D. melanogaster* ortholog of *GF15058* is *CG10178*. We found *CG10178* to be subsequently downregulated after the cold shock in *D. melanogaster* (Figure S2D). The function of the gene was inferred from sequence or structural similarity to be “uridine diphosphate (UDP) glycosyltransferase activity” [47]. 

**Figure 6:**
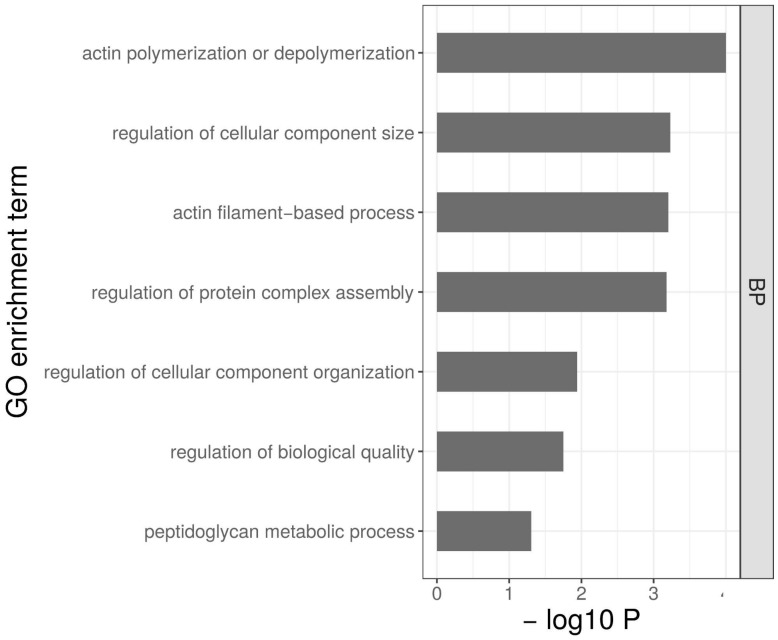
Gene Ontology enrichment analysis for genes that overlapped between the following two categories: (1) higher expressed before the cold shock (at room temperature) in the cold-tolerant fly strains than in the cold-sensitive fly strains, and (2) upregulated in the cold-sensitive fly strains at 90 min after the cold shock. Gene Ontology terms are plotted according to the significance of their enrichment (−log10 *p*-value after Benjamini–Hochberg correction). Enrichment was only significant in terms of the category biological process (BP).

With the second approach, we analyzed the overlap of genes that were upregulated in the cold-tolerant phenotype already at room temperature with those genes that were upregulated in the cold-sensitive phenotype only after the cold shock. At room temperature, 552 genes had higher expression in the cold-tolerant phenotype compared to the cold-sensitive phenotype. At the time point 15 min after the cold shock, 82 genes were upregulated in the cold-sensitive phenotype only. There was an overlap of 13 genes between these two groups. There was no significant GO enrichment in this overlap. Among these genes was the ortholog of *Jun-related antigen* (*Jra*). *Jra* showed a significant interaction of phenotype and cold shock at the 90 min time point in *D. melanogaster* [11]. In the late recovery phase (90 min after the cold shock), 1086 genes were upregulated in the cold-sensitive phenotype compared to the cold-tolerant phenotype. Among these genes were 100 genes that overlapped with the 552 genes that are upregulated in the cold-tolerant phenotype compared to the cold-sensitive phenotype at room temperature. This set of overlapping genes was significantly enriched for seven GO terms (Figure 6, Additional File 4: Table S15). The strongest enrichment was seen in the category “actin polymerization or depolymerization”, which was driven by six genes: *capping protein alpha* (*CPA*, *GF11927*), *capping protein beta* (*CPB*, *GF20820*), *actin-related protein 2/3 complex*, *subunit 3B* (*Arcp3B*, *GF21827*), *actin*-*related protein 2/3 complex*, *subunit 4* (*Arcp4*, *GF14506*), *twinstar* (*GF13484*), and *twinfilin* (*GF16237*).

## 4. Discussion

We assessed CCRT in two populations of *D. ananassae*. Interestingly, a tropical population originating from the ancestral species range showed two segregating phenotypes: cold-tolerant (fast CCR) and cold-sensitive (slow CCR) fly strains. With the aim to uncover candidate genes for cold adaptation in *D. ananassae*, we analyzed differential gene expression in response to a cold shock in the two phenotypes of this population. Note that we called differentially expressed genes using corrected *p*-values, and did not apply a log2 fold change cut-off. There is a detectable transcriptional response already early in the recovery phase (15 min after the cold shock). In both phenotypes, the strongest upregulation is seen in Hsps, headed by *Hsp70*. This is consistent with previous results reporting *Hsp70* to be upregulated in response to cold stress in *D. melanogaster* [11,48,49] and other insects [50], and emphasizes an early onset of a general stress response in both of the phenotypes. However, there is a remarkable difference in the number of differentially expressed genes, as about 10 times more genes are downregulated in the cold-sensitive phenotype. There is no significant GO enrichment at this set of differentially expressed genes, but it includes several genes associated with metal ion binding (Additional File 4: Table S6). As metal ion homeostasis is crucial for insect cold hardiness e.g., von Heckel et al. [11], MacMillan et al. [21], Storey and Storey [51], a lag in the transcription of such genes may have contributed to a prolonged CCR in the cold-sensitive flies. However, the reduced variation in gene expression in the cold-tolerant strains suggests that the adaptive potential arises from the elimination of maladaptive plasticity. While we did not see a general pattern of canalization in gene expression in the cold-tolerant strains as it was found in other drosophilids (*D. melanogaster* [11], *D. serreata* [52]), such a pattern might become more apparent when comparing gene expression in response to cold across multiple populations.

Later in the recovery phase (90 min after the cold shock), about 8% of all of the protein-coding genes were found to be upregulated, and 5–7% were found to be downregulated in both cold-tolerant and cold-sensitive strains (Table 1), which is slightly less than in *D. melanogaster* (9% and 10–11%, respectively [11]). Note that we used the same FDR cut-off (0.05) for both datasets (see also the Methods, Section 2.6). In both phenotypes, the transcriptional response at this time point is characterized by a continued upregulation of Hsps, an upregulation of genes involved in signaling and cell communication, and a downregulation of multiple GO terms that are related to various metabolic processes, which is, again, consistent with previous findings [11,53].

One of our central questions was: which genes differ in their response to the cold shock between the two phenotypic groups? The two distinct phenotypes were present as standing genetic variation within a single population, which is why we expected to find only a small number of candidate genes to underlie the difference in CCRT. Indeed, we found only two genes with a significant interaction between phenotype and cold shock: *GF14647* and *GF15058*. The function of the first gene (*GF14647)* is inferred from sequence or structural similarity to code for a protein that catalyzes the methylation of selenocysteine into Se-methylselenocysteine [47]. Se-methylselenocysteine has recently attracted attention in cancer research, as it was shown to provide organ-specific protection against chemotherapy-induced toxicity in rats [44]. Interestingly, it was found to inhibit apoptosis in human neuroblastoma cells [54]. We found *GF14647* transcripts to be cold-inducible in both phenotypes of *D. ananassae*, but its upregulation persisted longer in the fast strains (Figure S2A). This might be of special interest, because we found evidence for the enrichment of apoptosis genes in the cold-sensitive strains at 90 min after the cold shock. Thus, we propose *GF14647* as an interesting new candidate gene whose role in cold tolerance should be investigated in more detail. The function of the second gene that showed a significant interaction (*GF15058)* is predicted to code for a protein with UDP-glycosyltransferase activity [47]. UDP-glycosyltransferases (UGTs) are membrane-bound enzymes that are located in the endoplasmatic reticulum. They catalyze the addition of a glycosyl group from a uridine triphosphate (UTP) sugar to a small hydrophobic molecule. Therefore, UGTs play an important role in maintaining homeostatic function and detoxification, and are known as major members of phase II drug metabolizing enzymes [55]. The cold shock led to a downregulation of *GF15058* in the cold-sensitive strains, but not in the cold-tolerant strains. Thus, we propose *GF15058* as a second new candidate gene for cold tolerance in *D. ananassae*. 

### 4.1. Delayed Onset of Apoptosis May Improve Recovery from Chill Coma

Comparing differential gene expression between cold-sensitive and cold-tolerant strains at the 90 min time point, the most striking pattern was the roughly fivefold enrichment for the GO term “regulation of apoptotic process” in the cold-sensitive strains, but not in the cold-tolerant strains. This may seem to be a consequence of the weaker cold hardiness in the cold-sensitive strains, and an effort of the organism to safely eliminate affected cells, thereby protecting the fly from further damage. However, it has been proposed that a cold-induced onset of apoptosis could in fact be the cause for a reduced tolerance toward cold stress. In this case, the apoptotic pathway is overactive, leading to an unnecessary death of cells that could have survived [56,57,58]. Thus, the reduced expression of genes that positively regulate the apoptotic pathway could provide an advantage that leads to faster CCR. 

### 4.2. Increased Expression of Hsps Is Unlikely to Improve Chill Coma Recovery Time in Drosophila

Hsps are found in all organisms, and are known for their essential role in maintaining protein homeostasis in various stress situations. They act as chaperones, and thereby assist in folding newly synthesized proteins, but also assist in the repair or degradation of proteins [59,60]. Hsps are subdivided into different classes based on their molecular weight. Small Hsps (sHsps) have been suggested to protect cells from oxidative stress, as their expression levels not only increase with heat or cold stress, but also with age [61,62]. Further, the knockdowns of two sHsps (*Hsp22*, *Hsp23*) have been linked to prolonged CCR [63,64]. In our analysis, we find Hsps to be the most strongly upregulated genes in both phenotypes and at both time points in the recovery phase. The transcriptional response after cold shock is stronger in the cold-sensitive strains than in the cold-tolerant strains for all of the sHsps. We see this effect for both *D. ananassae* (Figure 5A) and *D. melanogaster* (Figure 5B). Among the heavier Hsp classes, we see the same effect for *Hsp70* in both species. In *D. ananassae*, we see this effect also for most of the remaining Hsps. Transcriptional upregulation of *Hsp70* in response to cold stress has been reported for *D. melanogaster* [48,49] and other species [50]. Indeed, *D. melanogaster Hsp70* null mutant larvae showed a decreased survival rate after severe cold exposure [65], and knockdown of *Hsp70* was associated with reduced cold-hardiness in the pupae of the flesh fly *Sarcophaga crassipalpis* [66]. However, recent studies in adult flies rather suggest that the upregulation of *Hsp70* does not affect CCRT, but is rather related to repairing cell damage [11,67,68]. This is in line with our findings, which show that the upregulation of this gene is stronger in the cold-sensitive strains of both *D. melanogaster* and *D. ananassae*. Overall, our results suggest that an increase in expression of Hsps does not lead to an increase in cold tolerance, but rather reflects the degree of damage in the flies. 

### 4.3. Genes Involved in Actin Polymerization May Have a Preemptive Role in Cold Tolerance

We also considered transcriptional differences between cold-tolerant and cold-sensitive lines that were already present at room temperature. In total, 100 genes overlap between significantly upregulated genes at room temperature in cold-tolerant lines and significantly upregulated genes at 90 min after the cold shock in the cold-sensitive lines (Additional File 4: Table S11). The group of these genes was significantly enriched for “actin polymerization or depolymerization”. This enrichment was driven by six genes involved in actin polymerization: *CPA*, *CPB*, *Arpc3B*, *Arpc4*, *twinstar,* and *twinfilin*. *CPA* and *CPB* code for two subunits of the actin capping protein heterodimer, which binds at actin filaments and regulates their polymerization by inhibiting the addition and loss of actin [69]. In *D. melanogaster*, there was a slightly higher expression of *CPB,* but not *CPA,* in the cold-tolerant phenotype without cold shock [11]. The actin-related 2/3 protein complex is responsible for the de novo nucleation of actin filaments [70]. Genes related to that complex were recently shown to be upregulated in the coral species *Acropora muricata* when subjected to cold stress [71]. *Twinfilin* was found to play a crucial role in the maintenance of cytoskeleton actin dynamics in *D. melanogaster* [61]. *Twinstar* is also involved in actin polymerization. Recently, it was shown that knockdown of *twinstar* is associated with cell death in *D. melanogaster* [62]. In the porcelain crab *Petrolisthes cinctipes*, the homolog of *twinstar* (*cofilin)* was upregulated in response to heat stress [72]. Overall, the actin cytoskeleton was shown to play a central role in the cold hardiness of various species, including animals (e.g., *D. melanogaster* [11,73], mosquitos [74], crickets [14,15], zooplankton [75], silkworm [76]) and plants (e.g., alfalfa [77], pear [78]). Our results in *D. ananassae* support the important role of actin polymerization in cold hardiness. The identification of these six genes involved in actin polymerization and other genes with a significantly higher a priori expression in the cold-tolerant flies compared to the cold-sensitive flies (Additional File 4: Table S11) suggest that genes in this set may have a preemptive role, and that increased expression levels in these genes could have a direct effect on CCRT in this species. However, further work is required in order to functionally validate these findings and thus verify their biological relevance.

### 4.4. Strengths and Limitations of this Study and Outlook

We undertook the present study with an ancestral population of *D. ananassae*. Therefore, we do not have a direct means to conclude that our findings can be extended to other populations. Nevertheless, by using a single population with two segregating CCRT phenotypes, we were able to uncover gene expression differences between the two phenotypes without the confounding factor of population divergence. If we compared a tropical and a temperate population, many expression differences may reflect population divergence, both neutral and adaptive, which might not be related to our phenotype of interest. Such population comparisons can be done in the future, and the results should be interpreted in the light of our current results. Recently, MacMillan et al. colleagues reported 29% of all genes in *D. melanogaster* to be differentially expressed after cold acclimation for five days at 6 °C [53]. Their estimates are higher than the total number of differentially expressed genes that we identified for *D. melanogaster* after a short recovery time following a seven-hour cold shock without previous acclimation (20%). We consider this difference plausible, considering the different experimental setups. 

It needs to be pointed out that our phenotype of interest (CCR) is only one of several metrics that can be applied to determine insect cold hardiness in the laboratory. The test for CCRT was our method of choice, as we found this phenotype to segregate within the ancestral population, and we wanted to understand the transcriptional basis for it. It is a non-lethal test that allowed us to directly compare the two phenotypes (fast and slow CCR), as a standardized cold shock at the same temperature is applied. Furthermore, it also allowed us to directly compare our results to the previous study in *D. melanogaster*. However, while CCRT has been reported as being shorter in temperate species as compared to tropical ones, recent studies question the ecological relevance of this trait [9]. Temperature fluctuations may simply not be relevant for the tropical Bangkok population, as prevalent temperatures are mostly constant. Nevertheless, we suggest considering that the ability to cope with fluctuating temperatures could have been indeed an ecologically relevant trait for flies of this population when they migrated and started to colonize regions such as Kathmandu, where night temperatures can drop down to 0 °C. However, other metrics such as chill coma temperature or lower lethal temperature may be better predictors of cold hardiness in drosophilids [4,8,79]. Generally, the most relevant tests are those that measure actual differences in fitness such as fecundity, sperm count, or population growth [1]. 

We further want to point out that we measured levels of mRNA abundance, and therefore cannot directly refer to protein expression. As there is only a partial correlation between mRNA abundance and protein abundance, changes in protein accumulation and mRNA accumulation may not necessarily show the same pattern (as reviewed by Vogel and Marcotte in [80]). In addition, many of the significantly differentially expressed genes that we identified in our study changed transcript levels by less than twofold (Additional File 4). The biological relevance of such subtle changes in transcript abundance should be interpreted with care. Moreover, post-translational modifications such as protein phosphorylation are important mechanisms to tune stress responses [81], and may have contributed to the difference in CCRT.

For our study, we isolated RNA from whole flies instead of single tissues or organs. On the one hand, this was the first transcriptomic comparison of *D. ananassae* cold tolerance, and we had no a priori expectation regarding which tissues are most important for cold tolerance. We assumed that a cold shock induces a general physiological response affecting the entire organism. On the other hand, however, as suggested previously [11,14], it may be that expression differences between single tissues are masked in our data. Moreover, as we analyzed the transcriptome, mRNA levels were used as a proxy to infer a relation between differential expression and a difference in the phenotype. Therefore, it is not possible to establish a direct link between genotype and phenotype from our data. Some of our results may allow for a careful interpretation in the context of cause and effect. For example, the stronger response of Hsps in the cold-sensitive flies compared to the cold-tolerant flies suggests that their expression levels more likely reflect the degree of intrinsic stress, as a consequence of the weaker cold-hardiness in the cold-sensitive flies, rather than being the underlying, adaptive cause itself. Without functional evidence, it is not possible to differentiate these two possibilities. Hence, further work is required to validate the candidates identified in the present study. Such experiments may include screens for polymorphisms in the regulatory sequences of the candidates. Recent advances in genome editing now make it possible to study the effects of single nucleotide variants in detail [82]. This may be of particular importance, as the identified candidates are involved in crucial mechanisms such as cell death and actin polymerization, where gene knockdowns may be lethal.

## 5. Conclusions

We show that a tropical population of *D. ananassae* that originated from the ancestral species range contains substantial variation in cold tolerance. Our data suggest two different ways that adaptation to cold environments may have acted on the gene regulatory level in this species and population: (1) counteracting an overshooting apoptotic pathway, and (2) stabilization of the actin cytoskeleton. These two mechanisms likely represent general effects that are common in drosophilids [11,56,57] and other eukaryotes [44,58,71,83]. In addition, a number of unidentified genes (e.g., *GF14647* and *GF15058*) may be important, and could have adaptive potential for cold tolerance. Identifying their function could provide insights into additional cold tolerance mechanisms. Therefore, we suggest that experimental tests designed for elucidating the function of these unidentified genes should be carried out in order to validate their contribution to cold adaptation. These tests should include genome editing methods such as CRISPR/Cas9 in combination with additional measures of cold tolerance to assess the impact of the candidate genes on the fly’s fitness. Applying such approaches will not only help in understanding cold tolerance in natural populations of drosophilids, and other insects, but also uncover genes that have been subject to adaptive evolution, which in turn may give insights into general evolutionary processes driving adaptation over short and long timescales.

## Figures and Tables

**Table 1:** Differentially expressed genes in *D. ananassae*.

Timepoint	Direction	Fast CCR	Slow CCR	Fast vs. Slow
control	up	-	-	552 (3.87%)
	down	-	-	557 (3.91%)
15 min vs control	up	46 (0.32%)	82 (0.67%)	0
	down	11 (0.08%)	96 (0.78%)	0
90 min vs control	up	1096 (8.00%)	1086 (7.80%)	3 (0.02%)
	down	653 (4.80%)	986 (7.10%)	0
90 min vs 15 min	up	1114 (8.00%)	1145 (8.20%)	-
	down	733 (5.20%)	666 (4.80%)	-

Differentially expressed genes (false discovery rate, FDR = 5%) in *Drosophila ananassae* at room temperature (control) and 15 min and 90 min after a cold shock of three hours at 0 °C among cold-tolerant (fast CCR) and cold-sensitive (slow CCR) fly strains. Differentially expressed genes were analyzed with Deseq2; percentages were calculated according to the number of genes with non-zero read counts in the respective category (14,250 in total). See Additional File 4 for lists of upregulated and downregulated genes, including log2 fold changes.

**Table 2:** Differentially expressed genes in *D. melanogaster.*

Timepoint	Direction	Fast CCR	Slow CCR	Fast vs. Slow
control	up	-	-	1080 (8.10%)
	down	-	-	1075 (8.10%)
15 min vs control	up	96 (0.73%)	142 (1.00%)	0
	down	86 (0.65%)	97 (0.72%)	1 (0.01%)
90 min vs control	up	1258 (9.30%)	1257 (9.46%)	10 (0.08%)
	down	1342 (9.90%)	1401 (11.00%)	13 (0.10%)
90 min vs 15 min	up	1060 (7.80%)	1086 (8.00%)	-
	down	582 (4.30%)	837 (6.20%)	-

Differentially expressed genes (FDR = 5%) in *Drosophila melanogaster* at room temperature (control) and 15 min and 90 min after a cold shock of seven hours at 0 °C among cold-tolerant (fast CCR) and cold-sensitive (slow CCR) fly strains. Read counts were obtained from von Heckel et al. [11], and differentially expressed genes were analyzed with Deseq2; percentages were calculated according to the number of genes with non-zero read counts in the respective category (13,285 in total).

**Table 3:** Genes with significant phenotype x cold shock interaction in *D. ananassae*.

	Log2 Fold Change 15 min vs. Control		Log2 Fold Change 90 min vs. Control		Significance of Interaction ^a^
Gene	Cold-Tolerant	Cold-Sensitive	Cold-Tolerant	Cold-Sensitive	Corrected *P*-Value ^b^
*GF14647*	0.9597	0.6600	1.7301	0.2751	7.79 × 10^−3^
*GF15058*	−0.2726	−0.7023	0.2298	−0.5815	9.35 × 10^−3^

^a^ Significance of interaction applies to 90 min vs. control. ^b^ *p*-values were corrected according to Benjamini–Hochberg.

## Supplementary Materials

The following are available online at http://www.mdpi.com/2073-4425/9/12/624/s1, Additional file 1: Figure S1: Experimental overview. Additional file 2: Figure S2: Read counts for genes with phenotype x environment interaction. Additional file 3: Phenotype data: CCRT in eight isofemale fly strains from Bangkok, Thailand. Additional file 4: Differentially expressed genes. Additional file 5: Tables S1–S15: GO enrichment in differentially expressed genes. Additional file 6: Figure S3: PCA with 250 genes and all genes in the data set.
